# A Critical Role for Proinflammatory Behavior of Smooth Muscle Cells in Hemodynamic Initiation of Intracranial Aneurysm

**DOI:** 10.1371/journal.pone.0074357

**Published:** 2013-09-02

**Authors:** Max Mandelbaum, John Kolega, Jennifer M. Dolan, Adnan H. Siddiqui, Hui Meng

**Affiliations:** 1 Department of Mechanical and Aerospace Engineering, State University of New York, Buffalo, New York, United States of America; 2 Department Pathology and Anatomical Sciences, State University of New York, Buffalo, New York, United States of America; 3 Department of Neurosurgery, State University of New York, Buffalo, New York, United States of America; 4 Departments Neurosurgery and Radiology, State University of New York, Buffalo, New York, United States of America; 5 Departments of Mechanical and Aerospace Engineering, Neurosurgery, and Biomedical Engineering, State University of New York, Buffalo, New York, United States of America; 6 Toshiba Stroke and Vascular Research Center, State University of New York, Buffalo, New York, United States of America; UT-Southwestern Med Ctr, United States of America

## Abstract

**Background:**

Intracranial aneurysm initiation is poorly understood, although hemodynamic insult is believed to play an important role in triggering the pathology. It has recently been found in a rabbit model that while macrophages are absent during hemodynamic aneurysm initiation, matrix metalloproteinases (MMPs) are elevated and co-localize with smooth muscle cells (SMCs). This study investigates whether SMCs play a mechanistic role in aneurysm initiation triggered by hemodynamics.

**Methods:**

Aneurysmal damage was induced at the basilar terminus via bilateral common carotid artery ligation in rabbits (n = 45, plus 7 sham controls). 16 ligated rabbits were treated with doxycycline to inhibit MMPs, 7 received clodronate liposomes to deplete circulating monocytes, and the rest received no drug. Effects of the treatments on aneurysm development were assessed histologically 5 days and 6 months after ligation. MMP production and expression of inflammatory markers by SMCs was monitored by immunohistochemistry and in situ hybridization.

**Results:**

Treatment with doxycycline attenuated aneurysmal development examined at 5 days and 6 months, suggesting that MMPs contribute to aneurysm initiation. However, systemic depletion of macrophages did not decrease MMPs or suppress aneurysmal development. Immunofluorescence showed that during aneurysm initiation MMP-2 and MMP-9 were distributed in SMCs, and *in situ* hybridization indicated that they were transcribed by SMCs. In regions of early aneurysmal lesion, SMCs exhibited decreased expression of smooth muscle actin and increased NF-κB and MCP-1 expressions.

**Conclusions:**

During aneurysm initiation triggered by hemodynamics, SMCs rather than macrophages are responsible for MMP production that is critical for aneurysmal lesion development. These SMCs exhibit proinflammatory behavior.

## Introduction

Initiation of intracranial aneurysms (IAs) is poorly understood because the event is rarely observed in patients and the histological progression has never been followed in human tissues. However, because IAs are preferentially located at bifurcation apices or on outer curvatures, it is believed that hemodynamic forces are a predisposing factor for aneurysm formation. [1], [2] Furthermore, *de novo* aneurysm formation has been noted to occur in association with increased flow: in patients experiencing either a compensatory flow increase as a result of carotid occlusion [3] or increased flow at the arterial pedicles feeding an ateriovenous malformation. [4] In rodent models, IAs will form in animals with induced hypertension and collagen crosslinking deficiency but not until one of the common carotid arteries is ligated. [5] Hence, IA formation likely includes some degree of hemodynamic perturbation.

Histopathology indicates that IAs develop through active remodeling that removes the internal elastic lamina (IEL) and thins the artery wall. The degenerative changes during this remodeling have been attributed to inflammatory infiltrates [2], [6], [7] because macrophages, which can produce large amounts of the matrix metalloproteinases (MMP)-2 and MMP-9, have been observed in the walls of well-developed aneurysms in human autopsy tissue and in animal models weeks to months following induction. [6], [8] A role for inflammatory infiltrates has also been suspected because, in aortic aneurysms, the lesions form in regions of disturbed flow with low WSS, which are prone to atherosclerosis, leukocyte adhesion and infiltration. [9] However, the hemodynamic environment for initiation of saccular aneurysms in the brain is quite different, most notably in that it is distinguished by high wall shear stress (WSS) [2], [10], [11], which is not conducive to leukocyte infiltration. Therefore, it is unclear whether macrophage infiltration causes IA formation or is merely a consequence of earlier IA-initiating changes.

To study IA initiation, we developed a rabbit model in which IAs occur in response purely to hemodynamic insult. [11]–[14] Bilateral common carotid artery (CCA) ligation causes a compensatory increase in flow in the basilar artery (BA) [15], elevation of WSS at the basilar terminus (BT) [11], and formation of a *de novo* aneurysm at the BT. [16] As early as 2 and 5 days after ligation, the BT shows obvious aneurysmal lesions, most prominently characterized by IEL loss, medial thinning (with mural cell apoptosis), and bulging of the vessel wall. [11], [17], [18] These lesions occur specifically where the vessel wall experiences high WSS above a certain threshold and flow is accelerating to create a positive gradient in shear stress along the streamline. [11] The severity of aneurysmal damage correlates with the magnitude of the inciting hemodynamics. [11], [16].

It is intriguing that, while MMP-2 and -9 are upregulated in areas of aneurysmal damage within 2 days of the flow-increasing surgery, inflammatory cells are scant and not localized to the damage sites. [12] The few macrophages and neutrophils that are present are exclusively in the adventitia, far from the damaged IEL. Meanwhile, MMPs co-localize with smooth muscle cells (SMCs) in the media. Based on this evidence, we hypothesize that the early destructive remodeling leading to aneurysm formation can be caused by inflammatory behavior of resident SMCs rather than inflammatory infiltrates. The present study tests whether MMPs are critically involved in the destructive remodeling observed during hemodynamic initiation of IAs by inhibiting MMPs with doxycycline. Doxycyline attenuates formation and growth of abdominal aortic aneurysms, [19] but aortic aneurysms initiate in regions of low WSS, exhibiting extensive inflammatory infiltration, and associating intimately with atherosclerotic lesions, [20] and so may result from a very different disease process. Thus, the contribution of MMPs in IAs warrants separate characterization. The present study also tests if macrophages are necessary for the initial aneurysmal damage and if SMCs exhibit inflammatory behavior, including MMP production, during IA initiation.

## Methods

### Ethics Statement

All procedures were approved by the Institutional Animal Care and Use Committee of the State University of New York at Buffalo (protocol #NSG22112Y “Hemodynamic Induction of Pathologic Remodeling Leading to Intracranial Aneurysms”, Hui Meng, principal investigator).

### Hemodynamic Induction of IA

**Figure 1:**
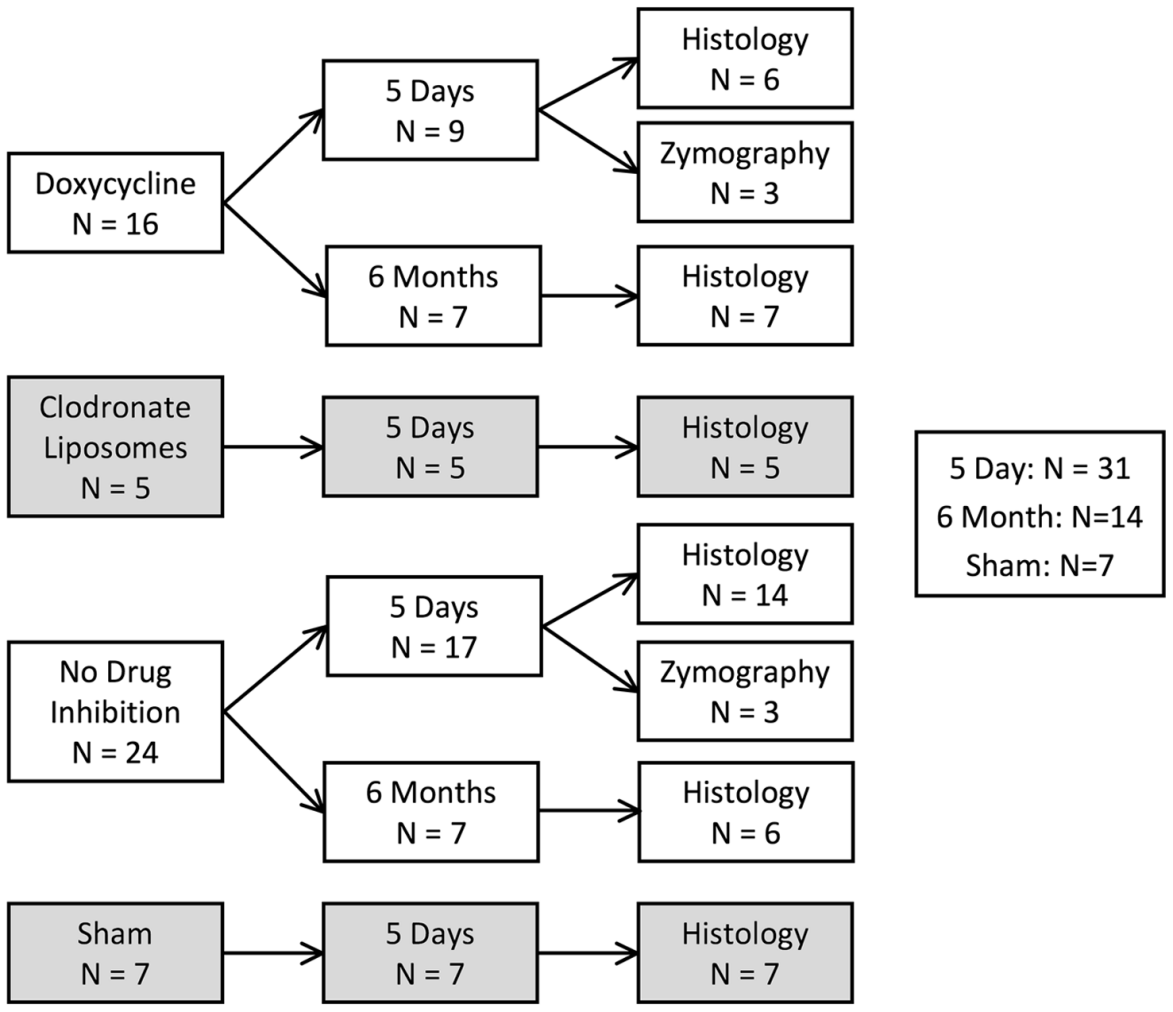
Flow chart of experimental rabbit groups used in the study.

IAs were induced at the BT (n = 45) as previously described. [16] Briefly, both CCAs were ligated in young, sexually mature (6–8 months old), unmated, female New Zealand White rabbits (4–5 kg) to create an immediate compensatory flow increase through the basilar artery without changing systemic blood pressure. [16] Aneurysmal damage, indicated by loss of the IEL, media thinning and bulge formation, occurs at the BT as early as 2 days after ligation. This pathological remodeling progresses over time with the most advanced lesions exhibiting large thin-walled bulges assuming the appearance of wide-necked low-aspect-ratio aneurysms at 6 months [13]. For all surgeries, anesthesia was induced with ketamine and xylazine and maintained with 2% isoflurane in oxygen. Animals were euthanized by intravenous administration of 100 mg/kg sodium pentobarbital followed by exsanguination through the jugular vein. Sacrifice occurred 5 days (n = 31) or 6 months (n = 14) after ligation, or 5 days after sham operations (n = 7) in which CCAs were surgically exposed but not ligated. [11], [21] Experimental groupings of animals are summarized in Figure 1.

### MMP Inhibition

The antibitotic, doxycycline, is a broad-spectrum MMP inhibitor at sub-antimicrobial doses, binding to MMP’s active zinc sites [22] and the inactive calcium ion site [23] to cause conformational changes and loss of activity. Doxycycline was used to inhibit MMPs in one group of rabbits (n = 16). Given the high oral bioavailability of doxycycline, [24] animals were given water supplemented with 20 mg/kg of doxycycline daily starting 5 days before ligation surgery and until sacrifice. In instances where animals did not drink enough water to receive the full dose (which sometimes occurred following surgery), remaining doxycycline dosages were given via intravenous injection. Both routes of administration result in comparable levels of circulating doxycycline. [24], [25].

### In Situ Gelatin Zymography

To assess MMP activity in tissues, *in situ* gelatin zymography was performed on rabbits sacrificed 5 days after flow-increase surgery (n = 3 in the doxycycline group; n = 3 in the non-doxycycline group). Immediately after sacrifice, the basilar bifurcation and BA were harvested, embedded in optimal cutting temperature compound, and quickly frozen. The tissue was then sectioned at a thickness of 10 µm. Zymography was performed by placing reaction buffer containing DQ gelatin (Molecular Probes) on tissue sections for 2 hours. Active MMP cleaves the DQ gelatin, unquenching fluorescence of fluorescein conjugated to the gelatin. The fluorescence of unquenched DQ gelatin was imaged immediately with an Axio Imager Z1 microscope (Zeiss) using excitation and emission wavelengths of 495 and 519 nm, respectively, and all fields were imaged under identical magnification, illumination, and exposure time. Fluorescence was then quantified on the digital micrographs using ImageJ software: First, background signal was determined by measuring the mean pixel values in an empty field in the image, and was subtracted from the entire image. Then, mean fluorescence in the tissue was calculated by manually outlining the entire vessel wall and measuring mean pixel intensity in this region. Analysis was performed only on frozen sections of the BA where all 3 layers of the vessel wall (intima, media, and adventitia) and an open lumen were discernable.

### Macrophage Depletion

Macrophages were depleted via intravenous injections of liposome-encapsulated clodronate, a cytotoxic agent (n = 7 for blood assays, but due to tissue disruption or folding during embedding/sectioning, only n = 5 were usable for histological analysis). When administered via the bloodstream, the liposomes become phagocytosed by circulating monocytes, which then die and are eliminated from circulation. [26] Clodronate liposomes were prepared as described by Van Rooijen and Sanders [26]. Rabbits received 15 mg/kg of liposome-encapsulated clodronate 5 days before surgery and again at the time of surgery. Macrophage depletion was confirmed by counting the number of circulating monocytes from blood drawn immediately before each dose and immediately before sacrifice.

### Histology

As previously described [13], the BA and BT were perfused via the vertebral arteries with phosphate-buffered saline immediately after sacrifice and pressure-fixed with 10% buffered neutral formalin for 30 minutes. Brains were removed to 10% formalin for 24 hours, after which the BT was excised, embedded in paraffin, and sectioned coronally through the entire bifurcation at a thickness of 4 µm (50–100 sections per specimen). After every 8 sections, the 9th and 10th sections were stained with Van Gieson and trichome stains, respectively, to provide an overview of the bifurcation.

For immunofluorescence analysis, sections were deparaffinized, rehydrated, and and boiled in 10 mM citric buffer (pH 6.0) for antigen unmasking. Sections were then stained with mouse monoclonal antibodies: anti-MMP-2 (Chemicon, 0.025 mg/ml), anti-MMP-9 (Chemicon, 0.02 mg/ml), anti- smooth muscle α- actin [SMA (AbCam, 0.5 µg/ml)], anti-calponin (AbCam, 0.025 µg/ml), anti-actin (AbCam, 0.048 mg/ml), anti-macrophage RAM-11 (Dako, 0.362 mg/ml), anti- NF-κB- p65 active subunit (Millipore, 0.01 mg/ml), or anti MCP-1 (AbD Serotec, 0.02 mg/ml). Primary antigens that were stained singly were visualized by indirect immunofluorescence using Dylight 647-conjugated goat anti-mouse antibody (Jackson ImmunoResearch). Fluorescence was imaged using a Zeiss Axio Imager Z1 microscope with excitation/emission wavelengths of 650 nm/668 nm.

To assess the presence of macrophages, sections were also incubated with 0.3% H_2_O_2_ for 5 minutes to inactivate endogenous peroxidases and stained with anti-macrophage RAM-11 (Dako, 0.362 mg/ml). Staining was visualized with an EnVision+ System-HRP (DAB) kit (Dako).

To stain sections for SMA, calponin, and total actin simultaneously, all of which use mouse monoclonal antibodies, sections were first stained with the SMA antibody labeled with Alexafluor 568 Zenon probe (Invitrogen) and the calponin antibody labeled with Alexafluor 647 Zenon probe (Invitrogen). The ratio of probe to antibody was 5 µL of probe per µg of antibody. Sections were then stained with a mouse monoclonal antibody to all actin isoforms directly conjugated to fluorescein (AbCam). Fluorophores were imaged separately using excitation/emission wavelengths of 495 nm/519 nm, 578 nm/603 nm, and 650 nm/668 nm for the fluorescein, Alexafluor 568 and Alexafluor 647 probes, respectively.

### Aneurysm Scoring

Sections from pressure-fixed BT tissues were scored for aneurysm formation as described in Metaxa et al., [11] with the aneurysm development score (ADS) defined in Meng et al. [13]. The sections that were scored for each animal were from the central aneurysmal lesion, or through the coronal midline of the bifurcation if no legion was evident. To identify the vessel midline, overall geometry was determined from the full set of trichrome-stained sections (approximately every 40 µm across the bifurcation). To locate lesions, the adjacent Van Gieson-stained sections were examined for regions where the IEL was disrupted. The section with the greatest length of IEL disruption was taken as the center of the lesion. In most cases, the central section was readily apparent by visual inspection because of obvious IEL loss that was distinctly greater than other sections. In cases where IEL loss was not clearly greatest in one particular section, the length of IEL loss was measured using ImageJ software [27] with consultation from 2 blinded observers. After identification of the central lesion or vessel midline, ADS was scored based on that pair of sections (Van Gieson- and Trichrome-stained) by two blinded observers. Using ImageJ, the observers measured the length of IEL loss, media thinning, and bulging. The percentage of media thinning was also quantified: the average thickness of the media was measured in the region of interest and then normalized by the average thickness of the media in healthy, downstream tissue in the same artery. Using these measures, we calculated aneurysmal destruction score by measuring the total length of regions where media thinning, bulge, and IEL loss simultaneously presented, multiplying it by the percentage of media thinning, and normalizing it by the diameter of the BA in the same animal.

### In Situ Hybridization

Frozen sections were stained for SMA using indirect immunofluorescence, then acetylated, treated with 20 µg/ml proteinase K (Sigma Aldrich) for 15 minutes, and incubated overnight at 53°C with digoxigenin-conjugated locked nucleic acid enhanced probes (Exiqon) for MMP-2 or MMP-9 (5′-TGTAACCATAGCGGAACAGGT-3′ for MMP-9; 5′-AAGGCTGATTAACTACGGAAGT-3′ for MMP-2). Probes were detected using sheep monoclonal antibody to digoxigenin (AbCam) and DyLight488-conjugated secondary (Jackson ImmunoResearch).

### Quantitative Assessment of Fluorescence Staining

Immunofluorescence was quantified on digital micrographs using Zeiss AxioVision Software. Analysis was performed on sections immediately adjacent to the trichrome- and Van Gieson-stained slides used to score aneurysmal damage. To minimize inter-slide variability, all specimens for any given staining were stained at the same time and imaged in a single session under identical conditions of magnification, illumination and exposure time, and within the linear dynamic range of the camera. Background signal was removed in each image by calculating the mean intensity of an empty field in the image and subtracting this baseline from the entire image. Staining intensities were then measured within desired regions of interest by manually outlining the regions using selection tools in the analysis software.

### Statistical Analysis

All values are expressed as mean ± standard error. Student’s t-test was used when comparing aneurysm measures between experimental groups; paired t-tests were used when comparing different regions of histological sections.

## Results

### Doxycycline Attenuated Hemodynamic Induction of IAs

**Figure 2:**
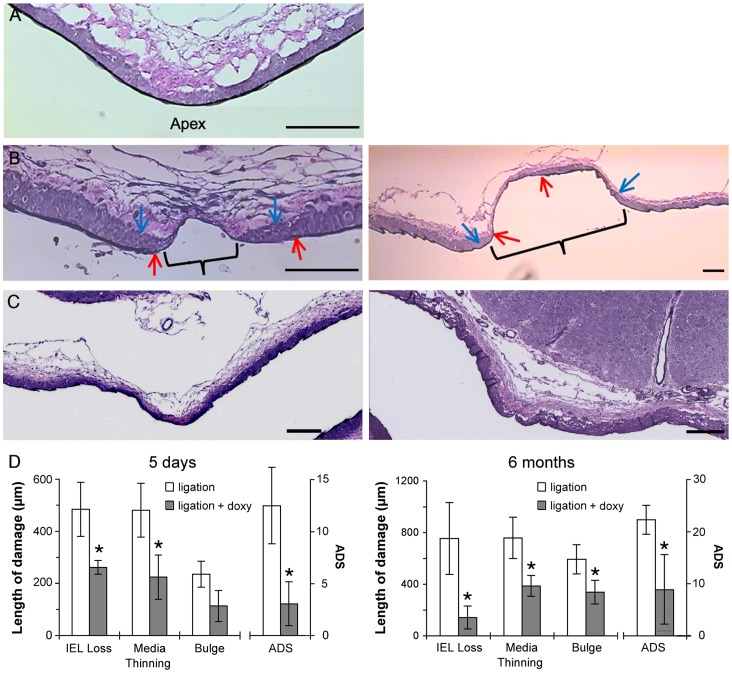
Inhibition of flow-induced aneurysmal damage by doxycycline. (A) Van Gieson staining of the BT 5 days after sham surgery shows dense, continuous IEL and uniform media. (B) BT 5 days (left) and 6 months (right) after bilateral CCA ligation, showing IEL loss (between red arrows), media thinning (between blue arrows) and bulge formation (bracket). (C) BT in doxycycline-treated rabbits 5 days (left) and 6 months (right) after ligation. IEL is largely intact and there is little medial thinning or bulge formation at either time. Scale bars in (A–C) = 100 microns. (D) The lengths of IEL loss, media thinning, bulging, and the ADS are all reduced 5 days after ligation compared to untreated animals (left) with all the differences except bulging being significant (* indicates p<0.05). All 4 parameters are significantly reduced after 6 months (right).

All experimental animals that underwent bilateral CCA ligation but were not treated with drugs exhibited aneurysmal damage at the BT (n = 24), with clear IEL loss, media thinning, and bulge formation 5 days after ligation and larger zones of media thinning and bulging after 6 months (Figure 2A, 2B). To test whether MMPs contributed to the damage, 16 rabbits were treated with doxycycline, a broad-spectrum inhibitor of MMPs. At the BT, doxycycline reduced the severity of aneurysm formation (Figure 2C, 2D). 5 days after ligation, IEL loss was significantly attenuated in doxycycline-treated animals compared to untreated animals (length of IEL loss was 262±26 µm vs. 484±104 µm, p<0.05). At 6 months, the difference was even greater (142±89 µm vs. 754±279 µm, p<0.05), with completely intact IEL in 3 of 7 treated animals. Aneurysmal bulging length was also reduced by doxycycline at 5 days (113±60 µm vs. 235±50 µm, p = 0.07), reaching statistical significance at 6 months (387±79 µm vs. 759±160 µm, p<0.05). Media thinning length was significantly reduced by doxycycline at both times (5 days: 224±85 µm vs. 481±103 µm, p<0.05; 6 months: 387±81 µm vs. 759±160 µm, p<0.05). The composite ADS was significantly decreased by doxycycline treatment at both times (3.6±2.7 vs. 12.2±3.8 at 5 days, p<0.05; 8.5±6.3 vs. 22.1±3.3 at 6 months, p<0.05).

**Figure 3:**
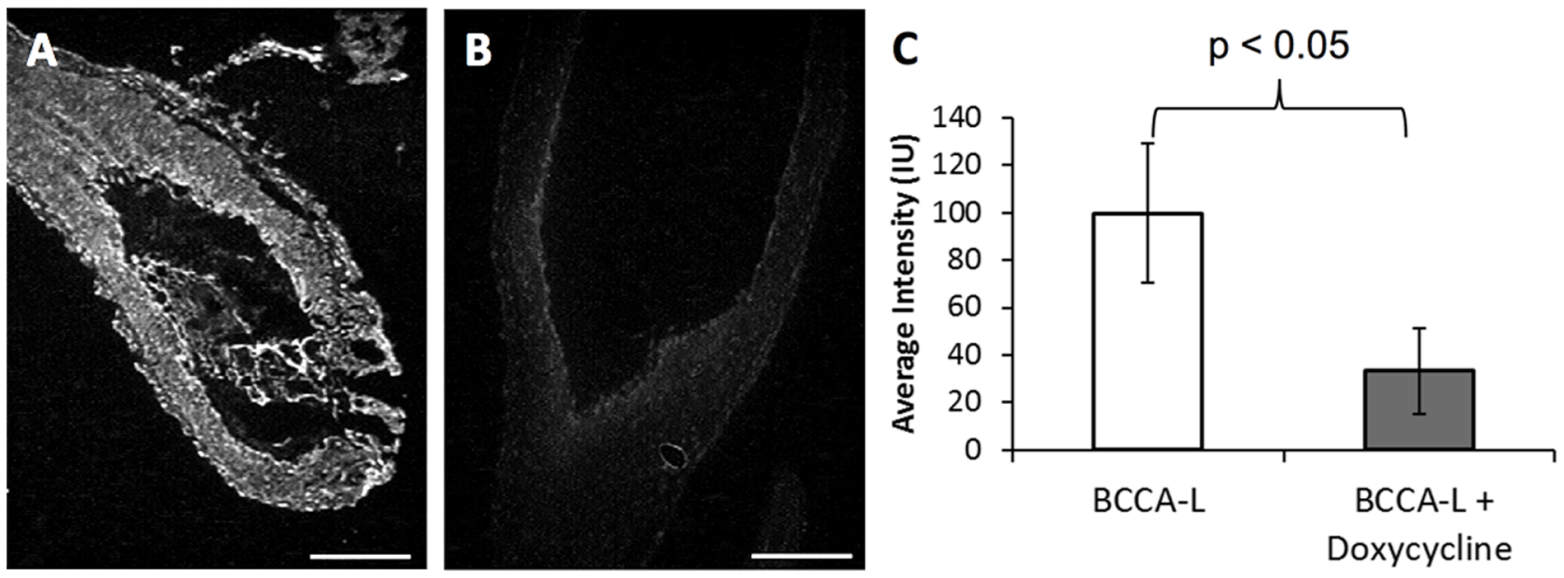
Inhibition of MMP activity by doxycycline. The BA was examined for gelatinolytic activity and MMP levels in ligated animals treated with or without doxycycline. (A) In situ zymography showing fluorescence of DQ-gelatin in the BA 5 days after carotid ligation. (B) Zymography of the BA 5 days after ligation in a rabbit treated with 20 mg/kg/day doxycycline. Scale bar = 50 microns. (C) Average intensity of unquenched fluorescence in the BA of ligated rabbits (n = 3 for each condition) with and without doxycycline (* indicates p<0.05). IU = intensity unit.

Inhibition of MMPs was confirmed by examining gelatinolytic activity in the BA by in situ zymography (Figure 3). After carotid ligation, the BA ordinarily exhibits increased MMP activity during the course of expansive remodeling to accommodate increased flow [28]. Zymography of frozen tissue sections taken 5 days after ligation showed high DQ-gelatin fluorescence, indicative of gelatinolytic activity, throughout the BA wall in untreated animals (Figure 3A), but very little fluorescence in doxycycline-treated animals (Figure 3B). The average fluorescence intensity as determined by quantitative image analysis was significantly reduced by doxycycline from 100±29 intensity units (IU) to 33±18 IU (p<0.05, n = 3) (Figure 3C).

### Macrophage Depletion did Not Suppress IA Initiation

**Figure 4:**
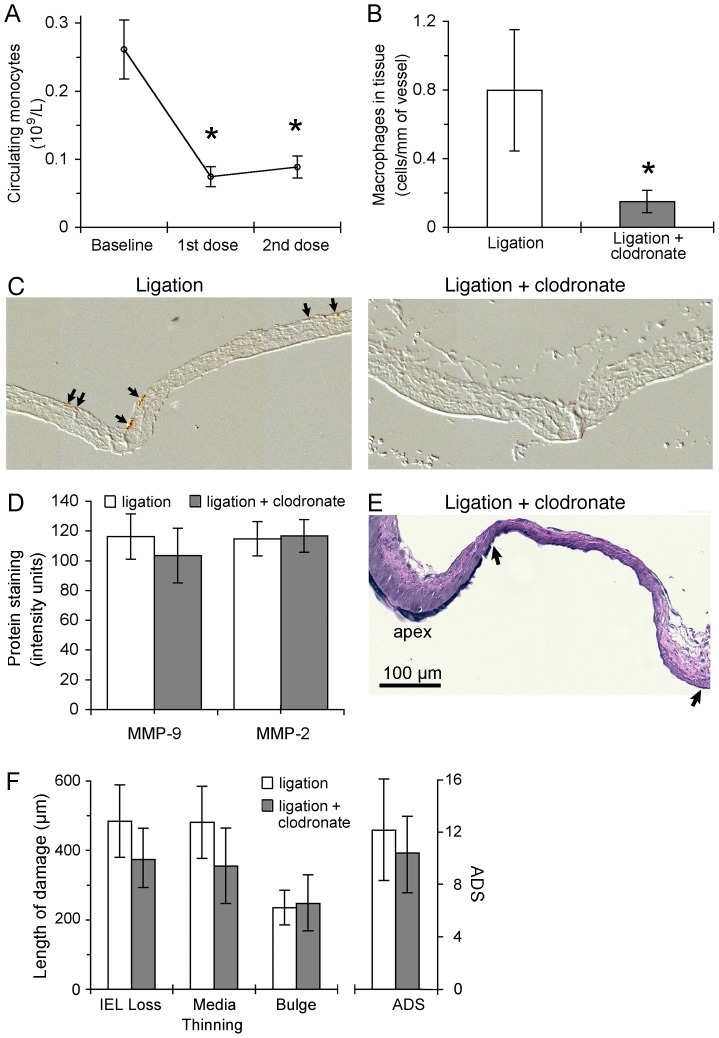
Effects of clodronate (monocyte depletion) on IA formation. (A) Clodronate liposomes reduced the number of circulating monocytes from 260±110 cells/µl to 70±40 cells/µl at the time of surgery, and the reduction was maintained through the experiment (90±40 cells/µl at sacrifice) (n = 7; * indicates p<0.05). (B) Clodronate liposomes also significantly lowered the number of adventitial macrophages detected by immunostaining of the basilar artery and bifurcation in ligated animals from 0.80±1.0 per mm of vessel (n = 8) to 0.15±0.15 (n = 5, * indicates p<0.05). (C) Immunohistochemistry with RAM-11 antibody shows the distribution of macrophages (brown color, arrows) at the BT in ligated animals (left) and their absence in ligated animals treated with clodronate liposomes (right). (D) Macrophage depletion by clodronate did not affect MMP levels in the media of the BT measured by immunostaining at 5 days. (E) Van Geison staining shows media thinning, bulge formation, and prominent IEL loss at the BT (between arrows) in a clodronate-treated rabbit 5 days after ligation. Bar = 100 (F) The length of vessel exhibiting IEL loss, media thinning, or bulge formation was not significantly different between ligated animals with and without clodronate, nor was the ADS significantly diminished by macrophage depletion (p≤0.05).

We tested the role of macrophages in IA initiation by depleting circulating monocytes in ligated animals via injection of clodronate liposomes. Clodronate liposomes reduced the number of circulating monocytes and lowered the number of adventitial macrophages detected by immunostaining (p<0.05) (Figure 4A–C). However, clodronate liposomes did not decrease the amount of MMP-2 (115±11 vs. 117±11 IU) or MMP-9 (116±15 vs. 103±18 IU) at the BT during IA initiation (Figure 4D), nor did it significantly reduce aneurysmal damage (Figure 4E, 4F). 5 days after CCA ligation, clodronate-treated rabbits were not significantly different from untreated animals in IEL loss (371±84 vs. 484±104 µm; p = 0.21), media thinning (340±110 vs. 481±104 µm; p = 0.19), bulge formation (249±89 vs. 236±50 µm; p = 0.45), and the composite ADS (10.2±3.2 vs. 12.2±3.8; p = 0.34).

### SMCs Expressed MMP-2 and MMP-9 during IA Initiation

**Figure 5:**
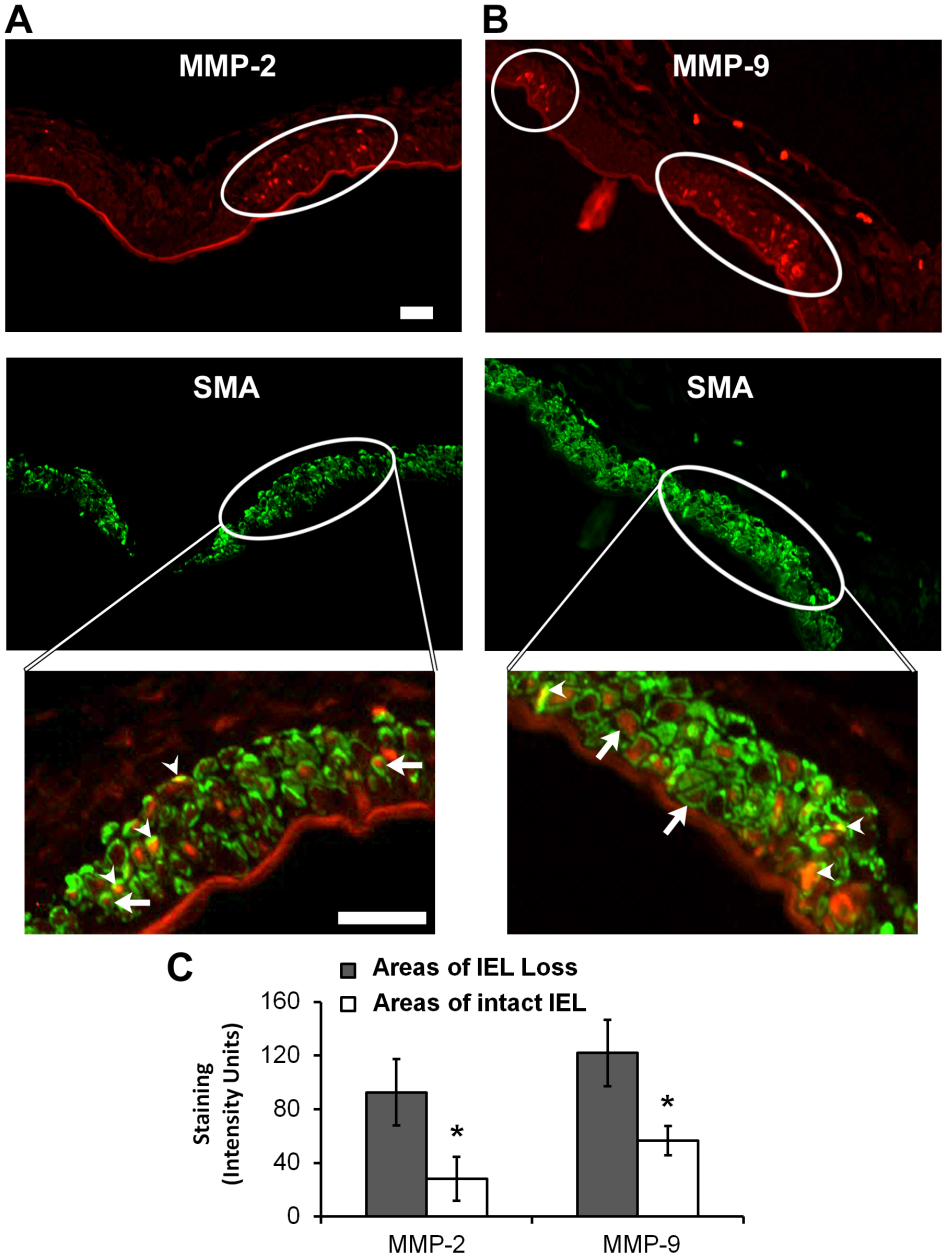
Localization of MMP expression during IA initiation. (A,B) The BT was stained for MMP-2 and -9 (top, red) and smooth-muscle actin, SMA, (middle, green) 5 days after CCA ligation. Ovals mark zones of IEL loss (determined from Van Giessen staining in adjacent sections), which showed the highest MMP intensity. High-magnification overlays of MMP and SMA staining in these zones (bottom) show both MMP-2 and -9 directly overlapping SMA (yellow or orange regions; arrowheads) and in large perinuclear patches entirely encircled by SMA-positive cytoplasm (arrows). (C) Quantitation of MMP-2 and -9 intensity in the media in areas of IEL loss compared to neighboring zones with intact IEL shows significantly higher intensity in areas of aneurysmal damage (n = 12; * indicates p<0.05). Scale bars = 50 µm.

**Figure 6:**
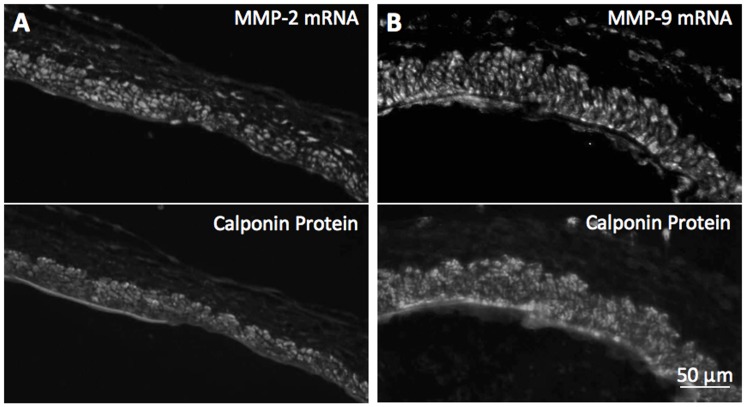
Expression of MMPs by SMCs in aneurysm initiation zones. (A, B). mRNA for MMP-2 and -9, respectively, detected by in-situ hybridization (top), with SMCs identified by counterstaining for calponin (bottom). mRNA for MMP-2 and -9 are expressed throughout the media in cells that stain for the SMC-marker, calponin. MMP mRNAs are also expressed in unidentified cells scatted through the adventitia that are not calponin-positive. Those cells may be fibroblasts. Scale bars = 50 µm.

Because macrophage depletion did not reduce MMP levels during IA initiation, we hypothesized that the MMPs associated with IA initiation come from an alternative source. However, the sites where IAs form are extremely small, so it was not possible to isolate enough material to measure MMPs biochemically and be able to assign a specific cellular origin. Therefore, in order to assess local differences in MMP levels we measured staining intensities in immunofluorescence images. Immunofluorescence showed MMP-2 and MMP-9 protein in SMCs, and higher levels in areas with aneurysmal change (Figure 5A, 5B). Quantitative analysis showed that both MMPs were significantly elevated in regions with IEL damage compared to intact areas with no aneurysmal changes (93±24 IU vs. 28±11 IU for MMP-2 and 122±25 IU vs. 57±16 IU for MMP-9; p<0.05) (Figure 5C). Furthermore, *in situ*-hybridization showed that mRNAs for both MMP-2 and MMP-9 co-localized with the SMC marker calponin (Figure 6), indicating that SMCs contained message for MMP-2 and MMP-9 synthesis during IA initiation.

### SMC Contractile Markers were Downregulated in Areas of Aneurysmal Damage

**Figure 7:**
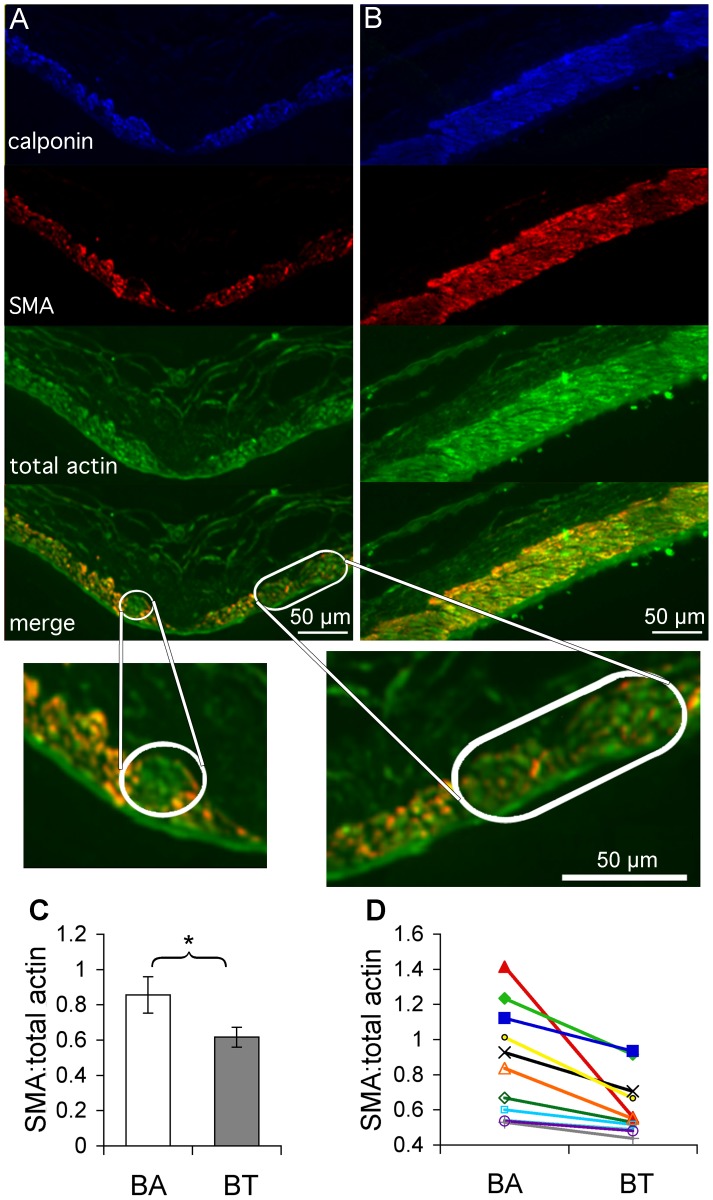
SMCs in aneurysmal regions exhibited a less contractile phenotype 5 days after ligation. BTs and BAs were harvested from 11 rabbits 5 days after ligation and stained for SMC markers. (A) Fluorescence staining for calponin (blue) identifies SMCs at the BT, which were also stained for SMA (red) and total actin (green). Overlay of SMA staining and total actin staining (bottom) shows reduced SMA (circled areas) in aneurysmal damage zones. (B) Analogous images for the BA. In contrast to the BT, SMA and total actin have very similar distributions. (C) The ratio of SMA to total actin was measured in SMCs at the BT and BA in 11 rabbits. The ratio was significantly lower at the BT. (D) All the BT and BA measurements are shown. In each animal (connected by a line), the ratio was lower at the BT than in the BA. Scale bars = 50 µm.

The increased expression of MMPs by SMCs suggested a deviation from their normal contractile phenotype. We examined markers of the contractile phenotype in SMCs during IA initiation. Compared to surrounding regions, SMCs in areas of aneurysmal damage expressed lower amounts of SMA and calponin (Figure 7A, 7B). Furthermore, the ratio of SMA to total actin was lower in the BT, where aneurysmal damage occurred, than in the BA, which experienced outward remodeling that was non-aneurysmal (0.65±07 IU vs. 0.86±0.10 IU, p<0.05 paired t-test, Figure 7C, 7D). In contrast, sham-operated rabbits had continuous strong expression of SMA and a consistent ratio of SMA to total actin throughout the local vasculature (0.96±0.06 IU at the BT vs. 0.92±0.08 IU at the BA).

### Markers of Inflammation were Upregulated in SMCs in Areas of Aneurysmal Damage

**Figure 8:**
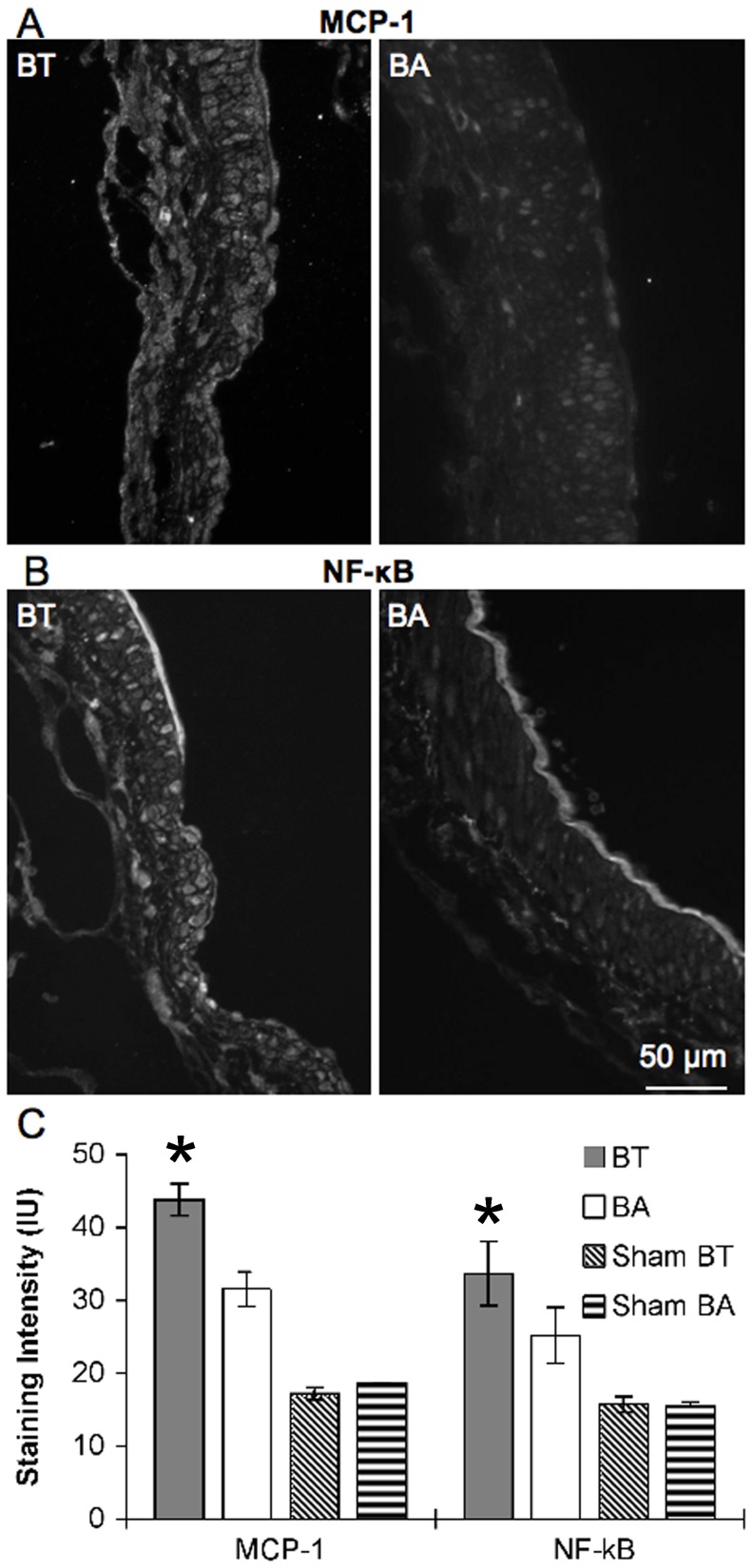
SMCs express increased inflammatory markers at the BT 5 days after ligation. BTs and BAs were harvested from 11 rabbits 5 days after ligation and from 7 rabbits 5 days after sham surgery, then stained for the inflammatory markers, MCP-1 and NF-κB. (A) Immunofluorescence of MCP-1 in an area of aneurysmal damage at the BT (left) compared to BA (right). (B) Immunofluorescence of NF-κB in an area of aneurysmal damage at the BT (left) compared to BA (right). (C,D) Average intensity of immunostaining shows that MCP-1 and NF-κB levels, respectively, are significantly higher in SMCs in the BT of animals after flow increase compared to the BA of the same animals and compared to levels in sham operated animals (* p<0.05). Scale bar = 50 µm. IU = intensity unit.

SMCs in regions of aneurysmal damage showed increased expression of MCP-1 and exhibited more staining with NF-κB-p65 antibody, which selectively binds to only the active form of NF-κB (Figure 8). Staining for total MCP-1 protein was higher at the BT in ligated animals vs. sham-operated animals (43.8±2.2 IU vs. 17±0.89 IU, p<0.05 via t-test ) and higher at the BT than at the BA after ligation (43.8±2.2 IU vs. 31.6±2.3 IU, p<0.05 via paired t-test). Activated NF-κB was significantly higher at the BT after CCA ligation than in sham-operated animals (33.7±4.4 IU vs. 15.8±1.0 IU, p<0.05 via t-test), and was also significantly higher at the BT than at the BA in the same animals (33.7±4.4 IU vs. 25.2±3.9 IU, p<0.05 via paired t-test).

## Discussion

Although it is generally accepted that hemodynamics play an important role in triggering aneurysm initiation, [29], [30] it is widely assumed that the ensuing IAs develop as a result of localized inflammatory infiltration that degrades the vessel wall, leading to thinning and weakening, ultimately producing the outward bulge that is an aneurysm. [17], [31], [32] The degradation is usually ascribed to matrix proteinases (especially MMP-2 and -9) produced by infiltrating leukocytes, which are commonly observed in mature human IAs and in animal models of IA. [6]–[8], [17] However, it is important to recognize that such observations of inflammatory infiltration have been made only after a fully developed aneurysm is evident (in the case of human specimens) or weeks after an aneurysm-inducing manipulation is made (in animal studies). Our studies are the first to capture IAs at the very beginning of lesion formation, and they indicate that inflammatory infiltrates are not necessary for IA inception.

In our hemodynamically induced aneurysms, early lesions are not endowed with inflammatory infiltrates. [12] The high WSS at IA initiation sites is likely not conducive for leukocyte infiltration, which requires sufficient blood residence time and endothelial adhesion and permeability. The latter conditions do occur under low and oscillatory WSS [9] and may develop after bulge formation to facilitate leukocyte infiltration later in IA development. Furthermore, in our system, macrophage depletion neither reduced local MMP production nor attenuated IA formation. Thus, for the initial aneurysmal damage that occurs in response to hemodynamic insult, an alternative explanation is needed.

We demonstrated that, in the early stage of hemodynamic IA development, resident SMCs produce MMPs. SMCs produced elevated levels of MMP-2 and -9 during the aneurysm-inducing hemodynamic insult well before bulging of the wall occurred, and did so specifically where the earliest IA damage was observed. Furthermore, this damage was inhibited by treatment with doxycyline. Although doxycycline is best known as an anti-microbial drug, it is a very potent inhibitor of MMP activity *in vitro* and *in vivo* at much lower doses [33] such as used in the present study. We cannot rule out doxycycline acting via another mechanism, but the drug did reduce MMP activity in the media of drug-treated animals (Figure 3), and it also suppressed loss of the IEL, a distinct matrix structure. We propose that SMC-derived MMPs are responsible for the initial aneurysmal damage, degrading IEL and thinning the media, in the absence of and independent of leukocyte infiltration. Such matrix-degrading behavior by SMCs could also facilitate the growth and subsequent rupture of “blister aneurysms”; a subset of rupture-prone aneurysms which have little to no inflammatory cell infiltrates. [34], [35].

We believe that inflammatory behavior of SMCs in response to hemodynamic insult could be an important driver of the initial damage in IAs. The initial damage may subsequently attract the inflammatory infiltrates observed in the human disease [6], [7] and in other animal models that employ additional instigating factors, such as hypertension and estrogen deficiency, whichare associated with increased vascular inflammation. SMCs in our early lesions express MCP-1, a chemoattractant for monocytes, and may also attract infiltrates through byproducts of matrix degradation. Such a response would amplify inflammatory damage in the wall and augment aneurysm progression. Inflammatory markers such as MMPs and NF-κB are also elevated in SMCs in human [36] and animal [8], [37] IAs, as in our current study. Furthermore, deficiency in MCP-1 or NFκB reduced aneurysm formation in hypertensive mice subjected to hemodynamic insult, while also reducing macrophage infiltration in the cerebral vessels. [37]–[39] The latter observations suggest that macrophage infiltrates are key inflammatory agents in the development of those IAs, but leave open the question of what stimulates the infiltration. Further understanding of how proinflammatory signals from SMCs contribute to recruitment of circulating leukocytes to the aneurysm wall is needed.

Phenotypic modulation of SMCs is not new to vascular pathology. SMCs lose some contractile traits and engage in matrix proteolysis in other instances of vascular remodeling. [40]–[43] SMC changes play an important role in atherosclerotic lesions, where SMCs upregulate NF-κB, [44] secrete cytokines [45], [46] and MMPs, [47] migrate into the intima, and proliferate to cause thickening of the wall and stenosis. [43], [48] SMCs in human IA tissue display reduced levels of desmin and adult myosin isoforms and elevated embryonic myosin, indicating reduced contractile phenotype. [36] In addition, our present study shows increased MMPs, NF-κB, and MCP-1 in SMCs of incipient IAs, suggesting a shift of SMCs toward inflammatory activities. A notable difference between SMCs in IA and in atherosclerosis is that the former undergo apoptosis [6] instead of proliferation, until the aneurysm wall becomes almost devoid of SMCs [21].

SMCs also shift phenotype in adaptive outward remodeling, when arteries enlarge to accommodate chronically increased flow. However, besides decreasing contractile markers [43] and increasing MMP production, SMCs in outward remodeling also synthesize new extracellular matrix [47] and proliferate at higher-than-normal rates to enlarge, rather than thin the media. [49] This again contrasts with the loss of SMCs in IAs. Thus, SMC behavior during IA initiation appears not to be simply a more extreme version of outward remodeling, because it involves cell death and matrix loss resulting in net tissue destruction rather than synthesis. It is interesting that in cerebral amyloid angiopathy, which like IA can lead to cerebral hemorrhage, SMCs exhibit increased contractile markers. [50] What causes vessel wall breakdown in amyloid angiopathy is unknown, but SMC dysfunction may be an important contributor.

It is not clear what causes SMCs to lose contractile proteins while becoming more proinflammatory during IA initiation. In our experimental system, IAs are induced solely by hemodynamic insult, and only endothelium directly experiences aneurysm-inducing hemodynamics (specifically, high WSS and positive WSS gradient) [11] when IEL loss and MMP upregulation are first observed. [12] Thus endothelial cells likely play a crucial role. In response to aneurysm-inducing hemodynamic stresses, they may produce paracrine signals that stimulate proinflammatory behavior in SMCs, including MMP secretion that degrades IEL and causes media thinning. The molecular responses of endothelia to the precise hemodynamics that induce IAs in vivo have not been fully characterized. One possible paracrine signal is interferon γ, a cytokine that downregulates contractile markers in SMCs, stimulates SMC apoptosis, and inhibits proliferation. [51], [52] Alternatively, hemodynamic insult may downregulate endothelial signals that maintain the normal SMC state. This occurs when endothelium is mechanically removed from an artery and the underlying SMCs lose contractile markers and undergo proliferation and migration contributing to stenosis. [53] Endothelial signals mediating IA initiation require further study.

## Conclusions

SMCs play a critical role in IA initiation in response to hemodynamic insult, producing MMPs that promote aneurysmal damage. It appears that macrophages are not necessary for IA initiation. Aneurysmal damage by SMCs involves conversion of SMCs from their quiescent contractile phenotype to a more inflammatory state. The induction of SMC inflammation during IA initiation probably involves signals from overlying endothelium. Further investigation of SMC inflammatory behavior and its regulation in IAs could lead to new prevention strategies or targets for treatment.
